# Determination of Alkali-Sensing Parts of the Insulin Receptor-Related Receptor Using the Bioinformatic Approach

**Published:** 2015

**Authors:** I. E. Deyev, N. V. Popova, A. G. Petrenko

**Affiliations:** Laboratory of Receptor Cell Biology, Shemyakin–Ovchinnikov Institute of Bioorganic Chemistry, Russian Academy of Sciences, Miklukho-Maklaya Str., 16/10, 117997, Moscow, Russia

**Keywords:** receptor, alkaline pH, phosphorylation

## Abstract

IRR (insulin receptor-related receptor) is a receptor tyrosine kinase belonging to the insulin receptor family, which also includes insulin receptor and IGF-IR receptor. We have previously shown that IRR is activated by extracellular fluid with pH > 7.9 and regulates excess alkali excretion in the body. We performed a bioinformatic analysis of the pH-sensitive potential of all three members of the insulin receptor family of various animal species (from frog to man) and their chimeras with swapping of different domains in the extracellular region. An analysis using the AcalPred program showed that insulin receptor family proteins are divided into two classes: one class with the optimal working pH in the acidic medium (virtually all insulin receptor and insulin-like growth factor receptor orthologs, except for the IGF-IR ortholog from *Xenopus laevis*) and the second class with the optimal working pH in the alkaline medium (all IRR orthologs). The program had predicted that the most noticeable effect on the pH-sensitive property of IRR would be caused by the replacement of the L1 and C domains in its extracellular region, as well as the replacement of the second and third fibronectin repeats. It had also been assumed that replacement of the L2 domain would have the least significant effect on the alkaline sensitivity of IRR. To test the *in silico *predictions, we obtained three constructs with swapping of the L1C domains, the third L2 domain, and all three domains L1CL2 of IRR with similar domains of the insulin-like growth factor receptor. We found that replacement of the L1C and L1CL2 domains reduces the receptor’s ability to be activated with alkaline pH, thus increasing the half-maximal effective concentration by about 100%. Replacement of the L2 domain increased the half-maximal effective concentration by 40%. Thus, our results indicate the high predictive potential of the AcalPred algorithm, not only for the pH-sensitive enzymes, but also for pH-sensitive receptors.

## INTRODUCTION

The insulin receptor (IR) family consists of IR, insulin- like growth factor receptor (IGR-IR), and the insulin receptor-related receptor (IRR). All three receptors are highly homologous receptor tyrosine kinases with a single transmembrane segment, which exist as homologous dimers linked via cystine bridges [1, 2]. This property makes the members of the IR family different than other tyrosine kinase receptors, which form non-covalent dimers only after activation. As they mature, both monomers are proteolyzed in the near-membrane zone of the extracellular portion. As a result, a receptor molecule consists of two pairs of covalently bound alpha and beta subunits.

All three receptors contain the leucine-rich L1 and L2 domains in the extracellular N-terminal portion of the alpha subunit with the C domain (furin-like cysteine- rich region) located between them. These domains are followed by three fibronectin repeats: FnIII-1, FnIII-2, and FnIII-3 [3]. The tyrosine kinase domain is located in the cytoplasmic portion of the beta subunit. The degree of homology between IGF-IR and IRR is somewhat higher than that between IR and IRR [2]; hence, it is believed that duplication and separation of the genes encoding IGF-IR and IRR were evolutionarily later processes compared to separation of the insulin receptor gene [2].

Since receptors are pre-dimerized, binding of the peptide ligand to the extracellular portion of IR or IGFIR causes changes in conformation, which result in autophosphorylation of the tyrosine residues located in the cytoplasmic tyrosine kinase domain. As opposed to its homologs, IRR has no ligands of peptide or protein nature. Meanwhile, we found that IRR is activated at pH of the extracellular fluid higher than 7.9 [4, 5]. *In vivo *experiments using mice with IRR gene knockout demonstrated that this receptor is involved in the regulation of renal excretion of excess alkali in the form of bicarbonate [6, 7]. Mapping of the regions determining the pH sensitivity of IRR has shown that several extracellular domains are responsible for receptor activation [5, 8], which ensures positive cooperation in activation (the Hill’s coefficient being ~ 2.4) [5, 9].

The following question is of obvious fundamental interest: what is the reason for such striking differences between the functions of IR and IGF-IR receptors, on the one hand, and IRR, on the other hand. In this study, we used the bioinformatic approach to perform a comparative analysis of the pH sensitivity of IRR receptor and other receptors belonging to the insulin receptor family. The AcalPred program [10], which was designed to predict pH values (either acidic or alkaline) that would be optimal for enzyme function based on its primary structure, allowed us to divide the IR family into two types: the “acid-dependent” proteins (almost all IR and IGF-IR orthologs) and the “base-dependent” proteins (all IRR orthologs). This approach has made it possible to estimate the relative contribution of individual domains in the extracellular portion of IRR. The predicted properties of IRR chimeras and the IGF-IR receptor were verified *in vitro *by determining their pH sensitivity.

## EXPERIMENTAL

**The sequences of insulin family receptors**

All the sequences of ectodomains of the insulin family* Bos taurus *(BosTau), *Canis familiaris *(CanFam), *Cavia porcellus *(CavPor), *Coturnix japonica *(CotJap), *Danio rerio *(DanRer), *Equus caballus *(EquCab), *Felis catus* (FelCat), *Gallus gallus *(GalGal), *Gasterosteus aculeatus* (GasAcu), *Homo sapiens *(HomSap), *Macaca mulatta* (MacMul), *Microcebus murinus *(MicMur), *Monodelphis domestica *(MonDom), *Mus musculus *(MusMus),* Ochotonas princeps *(OchPri), *Oryctolagus cuniculus* (OryCun), *Pan troglodytes *(PanTro), *Rattus norvegicus* (RatNor), *Scophthalmus maximus *(ScoMax), *Sus scrofa* (SusScr), and *Xenopus laevis *(XenLae) were taken from the material accompanying this article [11]. Since the genes of the IR and IGF-IR receptors in *Danio rerio* are duplicated, additional symbols, either a or b, were used for them.

**Production of chimeric receptors**

The sequences encoding human chimeric receptors were produced by polymerase chain reaction using the following primers: for L1C(IGF-IR) IRR-HA, 5'-CATCCCTTGTGAAGGTCCTTGCCCTAAAGAGTGCAAGGTAGGC and 5'-cccGGtACcTGTCACCTCCTCCAGTCGGTA, then 5'-gggGGTACCGAATTCATGAAGTCTGGCTCCGGAGGAG; for L2(IGF-IR) IRR-HA, 5'-CACAAGTGCGAGGGGCTGTGCCCGAAGGTCTGTGAGGAAGAAA and 5'-cccGGTACCCGTCACTTCCTCCATGCGGTAA, then 5'-ggg- GGTACCGAATTCATGGCAGTGCCTAGTCTGTGG. The correct sequences of the resulting constructs was verified by sequencing.

**Transfection of eukaryotic cells and receptor activation**

HEK293 cells were grown on a DMEM medium containing 10% of a fetal bovine serum, 1% of penicillin/ streptomycin and 2 mM *L*-glutamine under standard conditions (37Ѓ‹C and 5% CO_2_). The cells were transfected with pcDNA3.1 plasmids encoding IRR-HA or chimeric receptors using Unifectin-56 (UnifectGroup) according to the manufacturer’s recommendations. In 36-40 hours after transfection, the cells were left in a serum-free growth medium for 2– h under standard conditions. In order to test receptor activation and plot the activation curves, the receptor-expressing HEK293 cells after “starvation” in the serum-free medium were incubated in phosphate-buffered saline containing 60 mM Tris-HCl with the target pH value for 10 min at room temperature. The buffer was subsequently removed, and the cells were immediately lysed in 1× SDS-PAGE buffer.

**Western blot and construction of activation curves**

SDS-PAGE (8%) and Western blot analysis were carried out using the standard protocol described in [12]. The total amount of receptors was determined using rabbit serum against the cytoplasmic portion of IRR (anti-IR/IRR); rabbit serum against phosphorylated IRR (anti-pIR/IRR) was used to detect the phosphorylated form. Anti-IRR antibodies were produced and characterized at our laboratory [5]. HRP-conjugated goat anti-rabbit antibodies (Jackson ImmunoResearch) were used as secondary antibodies. The resulting blots were scanned; specific signals were processed using the ImageJ software. The signal transmitted from antibodies to phosphorylated IRR was normalized with respect to the signal transmitted from the antibody against the C-terminal portion of the IRR receptor. The normalized signals for each pH value (*n *≥ 3) were further processed in the GraphPad Prism 5 software using Hill’s equation (One site –Specific binding with Hill slope analysis). As a result of the interpolation analysis, the Hill’s coefficient and the half-maximal effective concentration of hydroxyl ions for the activation curve of chimeric proteins were calculated using the GraphPad Prism 5 software.

## RESULTS AND DISCUSSION

Receptor tyrosine kinase IRR exhibits a unique property to be activated in an alkaline extracellular medium. This property makes IRR stand out both among other members of the insulin receptor family and among most tyrosine kinase receptors that are activated by peptides or proteins. We wondered whether it was possible to predict this unique property of the IRR receptor using modern bioinformatic approaches. The recently described AcalPred program was originally developed for predicting pH values (either acidic or alkaline) that would be optimal for enzyme functioning based on its sequence. This program is now available online and is the most reliable option among the previously reported algorithms for predicting pH values optimal for enzyme functioning [10]. As a result, the relative probability of the fact that the protein “prefers” to function either in an alkaline or acidic medium was determined; the overall probability is equal to 1. This algorithm was developed for soluble enzymes: therefore, we used sequences of the ectodomains of IR family receptors from about 20 various organisms, from frog to man, and analyzed them using the AcalPred software (complete names of the organisms and their abbreviations are given in the Experimental section). We provide the results of an analysis of ectodomains of the human insulin receptor family as an example. Thus, human IR was classified as an “acidic” protein with a probability of 0.95 and as an “alkaline” protein, with a probability of 0.05. Human IGF-IR belongs to “acidic” proteins with a probability of 0.92 and to “alkaline” proteins with a probability of 0.08. Finally, there is a probability of 0.25 that human IRR is an “acidic” protein and 0.75 that it is an “alkaline” protein.

**Fig. 1:**
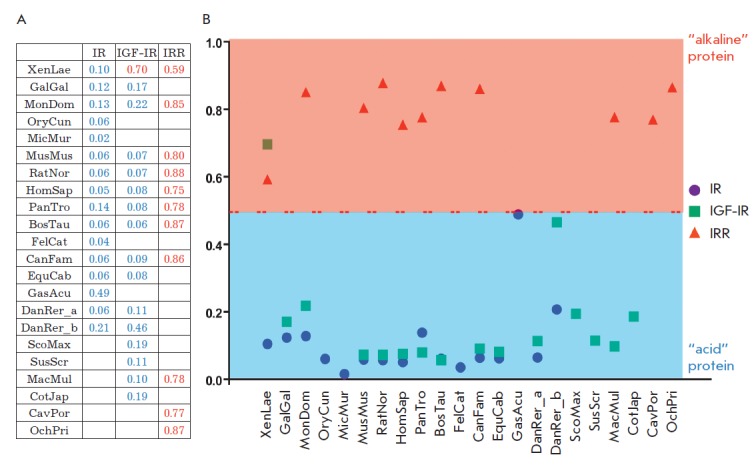
*A *– Sequence analysis of insulin receptor family ectodomains using the AcalPred program. The relative probabilities of activation of insulin receptor family ectodomains from various species at alkaline pH are shown in table. Full names of the species are given in the Experimental section*. B *– The graphical representation of the aforedescribed probabilities. The red line highlights the notional boundary value prediction – 0.5. Proteins with a predicted probability of alkaline sensitivity greater than 0.5 are shown as “alkaline” (in the red zone), and proteins with the probability less than 0.5 are indicated as “acidic” proteins (in the blue zone)

*Fig. 1A* shows the estimated probability that a protein is classified as an “alkaline” one for the rest of the ectodomains from different organisms. *Fig. 1B* provides a graphic interpretation of this Table; separation is made at a probability of 0.5 (“alkaline” proteins are placed above the line, while the “acidic” proteins are shown below the line). It is an interesting fact that the insulin receptor family is subdivided into two classes: a) IR and IGF-IR (except for frog IGF-IR), which are supposed to be “acidic” proteins; b) IRR orthologs, which are “alkaline” proteins. These results indicate that the AcalPred program can have a broader application than just analyzing the pH dependence of enzymes and can be used to predict alkaline activation and regulation of tyrosine kinase receptors. In particular, it is possible that the frog IGF-IR receptor, which was classified as an “alkaline” protein, can potentially be sensitive to a weakly alkaline environment.

**Fig. 2:**
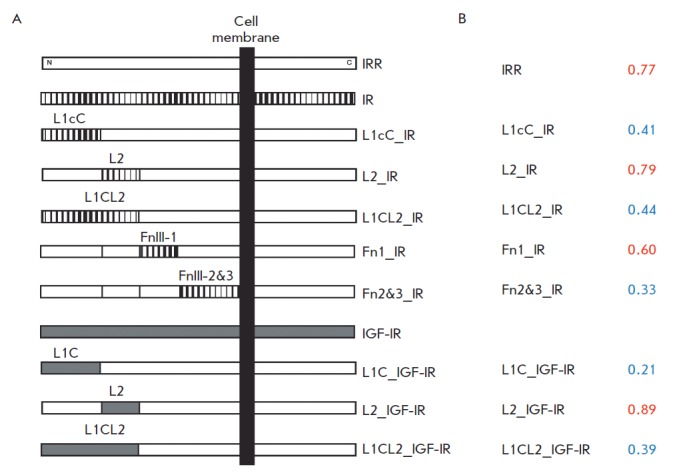
*A *– Schematic representation of the resulting chimeric proteins. IRR domains are shown in white; IR, in striped; and IGF-IR domains, in gray. L1 and L2 – L-domains, C – furin-like cysteine-rich domain, FnIII-1 and FnIII 2 & 3 – the first or second and third fibronectin repeats. *B – *Sequence analysis of the ectodomains of the chimeric receptors described above using the AcalPred program. The relative predicted probabilities that the ectodomain is an “alkaline” protein are shown in Table. Predicted probability values above 0.5 are shown in red (“alkaline” proteins), and those less than 0.5 are shown in blue (“acidic” proteins)

To evaluate the applicability of the AcalPred program for the analysis of the pH-sensitive properties of receptor tyrosine kinases, we experimentally compared certain properties of previously produced chimeric human IR and IRR proteins with replacement of some domains of the extracellular portion (*Fig. 2A*) [8, 9, 13]. Chimeric sequences were produced by replacing the first two L1C domains, the third L2 domain, all three L1CL2 domains, and the first fibronectin repeat FnIII-1 or the second and third fibronectin repeats in the IRR ectodomains with identical regions of the IR receptor.

An analysis of these sequences using the AcalPred program has demonstrated that replacement of the first two L1C domains or the second and third fibronectin repeats FnIII-2 and FnIII-3 is crucial for protein “alkalinity” (*Fig. 2B*). Replacement of the first fibronectin repeat FnIII-1 has a weaker effect; and replacement of the third L2 domain does not worsen the expected sensitivity to alkaline pH (*Fig. 2B*). These data show overall agreement with our experimental findings. Thus, it has been demonstrated that the substitutions with the strongest effect are the ones in the first two L1C domains or the second and the third fibronectin repeats FnIII-2 and FnIII, which are believed to form the main site of pH sensitivity in the IRR receptor [13]. The substitution of the L2 domain in IRR for an identical sequence from IR had a small but still noticeable effect on the sensitivity of IRR to alkaline pH [9], while no changes were predicted by AcalPred. The substitution of the first fibronectin repeat FnIII-1 for an identical IR fragment resulted in an effect stronger than that of the replacement of the L2 domain, comparable to the effect of L1C substitution but weaker than the effect of a replacement of the second and third fibronectin repeats FnIII-2 and FnIII-3 [9, 13], which showed agreement with the result predicted by the program. We can conclude that the AcalPred program has a predictive potential in analyzing chimeric receptors; however, it should be taken into account that the resulting parameters describe the probability rather than provide an accurate assessment of the pH dependence. In other words, the results are qualitative rather than quantitative.

**Table:** Hill’s coefficient (H) and half effect of hydroxyl ions (EC_50_) for the designated receptors

Receptor	Hill’s coefficient, H	Half effect, EC_50_, µM
IRR	2.4 ± 0.4	4.1 ± 0.4
L1C_IGF-IR	2.4 ± 0.6	9.9 ± 1.5
L2_IGF-IR	2.5 ± 0.4	5.8 ± 0.5
L1CL2_IGF-IR	1.6 ± 0.3	9.8 ± 2.6

**Fig. 3:**
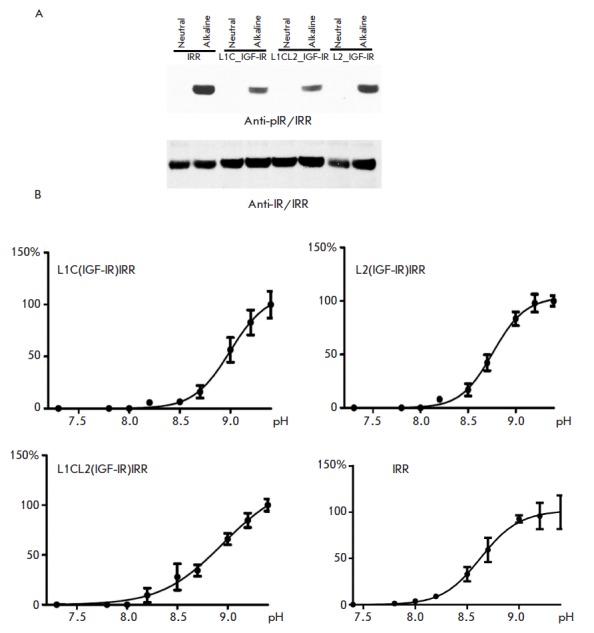
*A *– Activation of chimeric receptors at alkaline pH. HEK293 cells after expression of chimeric proteins were treated with 60 mM Tris-HCl buffers with pH 7.3 or 9.0, then lysed, and the proteins were separated by SDS-PAGE and transferred onto nitrocellulose membranes for Western blot analysis. An antibody to the phosphorylated IRR was used to detect phosphorylated receptors; the beta subunit was detected with an antibody against the C-terminal part of the IRR. *B – *pH-dependent activation curves of IRR and chimeric receptors. HEK293 cells after expression of chimeric proteins were treated with a buffer with pH ranging from 7.3 to 9.4 (7.3; 7.8; 8.0; 8.2; 8.5; 8.7; 9.0; 9.2; 9.4); the cells were then lysed, and the proteins were separated by SDS-PAGE and transferred onto a nitrocellulose membrane for Western blot analysis. Antibody to the phosphorylated IRR was used for detecting phosphorylated receptors; the beta-subunit was detected with an antibody against the C-terminal part of the IRR. The phosphorylated receptor was normalized to the total amount of the receptor (signal from beta-receptor subunits) for each pH value. The normalized signals for each pH (n ≥ 3) were calculated using the GraphPad Prism 5 software with One site – Specific binding with Hill slope interpolation. On each plot, the Y axis shows the percentage of the maximum average activation at pH 9.4

In evolutionary terms, IRR is structurally more similar to the IGF-IR receptor than to insulin receptor. Hence, in addition to analyzed IRR receptor where the L1CL2 domains were substituted for identical regions of the IGF-IR receptor [8], we produced two chimeric proteins where the L1C or L2 IRR domains were replaced with the corresponding domains of the IGFIR receptor (*Fig. 2A*). Next, we checked the response of the resulting receptors to increased pH of the extracellular medium. These proteins were expressed in HEK293 eukaryotic cells. The cells expressing chimeric receptors were treated with a buffer with pH 7.3 or 9.0. Same as IRR, L1C_IGF-IR, L1CL2_IGF-IR, and L2_ IGF-IR chimeras were activated in response to alkaline pH (*Fig. 3A*).

We plotted the curve showing the degree of activation of each chimeric receptor as a function of pH in a range from 7.3 to 9.4. The activation curves were recorded for all three chimeric proteins: L1C_IGF-IR, L1CL2_IGF-IR, and L2_IGF-IR (*Fig. 3B*). The Hill’s coefficient (H) and half effect of hydroxyl ions (EC_50_) were calculated for each receptor using the GraphPad Prism 5 software, which allows one to estimate the pH sensitivity of various chimeric receptors and cooperation of their interaction with an agonist. An analysis of the curves has demonstrated that when two L1C domains or one L2 domain are replaced, the Hill’s coefficient remains virtually unchanged. Thus, the Hill’s coefficient for L1C_IGF-IR was 2.4 ± 0.6 and 2.5 ± 0.4 for L2_IGF-IR and L2_IGF-IR, respectively, while 2.4 ± 0.4 for IRR. Meanwhile, replacement of the L1C domain increased the EC_50_ value by more than 100%, while substitution of the L2 domain increased it by approximately 40% (*Fig. 3B* and *Table*). Replacement of all three L1CL2 domains in the chimeric construct resulted in the strongest effect: the Hill’s coefficient decreased to 1.6 ± 0.3, while EC_50_ rose by more than 100%, up to 9.8 ± 2.6 µM (almost identically to the values in the chimeric construct with the first two L1C domains replaced) (*Table*) [8]. Such a decline in the Hill’s coefficient may be associated with the change in structure and mutual arrangement of pH-sensitive sites inside the ectodomain as the first three domains are replaced. Interestingly, replacement of the L1C and L2 domains in chimeric IRR receptors for the corresponding domains of insulin receptors led to more significant negative changes than insertion of IGF-IR domains [9]. Thus, replacement of L1C portions had the greatest negative effect, while the least effect was observed when the L2 domain was substituted.

## CONCLUSIONS

In this study, we used the bioinformatic approach to the analysis of the pH-sensitivity of the IRR receptor. The AcalPred algorithm elaborated to predict the optimal pH for the activity of soluble enzymes can also be used to describe the pH-sensitive properties of the members of the insulin receptor family. Moreover, this program can be employed to predict the contribution of individual structural fragments of the receptor to its pH-sensing function. It should be mentioned that the program mostly provides a qualitative result, while the quantitative conclusions may not be accurate enough.
